# Editorial: Novel Concepts in Cardiac Energy Metabolism: From Biology to Disease

**DOI:** 10.3389/fcvm.2019.00097

**Published:** 2019-07-12

**Authors:** Thomas Pulinilkunnil, Petra Kienesberger, Jeevan Nagendran

**Affiliations:** ^1^Department of Biochemistry and Molecular Biology, Faculty of Medicine, Dalhousie University, Halifax, NS, Canada; ^2^Department of Surgery, Faculty of Medicine and Dentistry, University of Alberta, Edmonton, AB, Canada

**Keywords:** energy metabolism, ATP, cardiomyocyte, heart failure, fatty acid

Energy metabolism is a process that is central to cardiac health and disease. High ATP turnover in the myocardium is required to maintain contractile function. ATP generation within the mitochondria involves oxidative decarboxylation of fatty acids, pyruvate, and the Krebs cycle to generate reducing equivalents for the oxidative phosphorylation of ADP in the electron transport chain. In the healthy adult heart, the majority of ATP is generated through the oxidation of fatty acids, and a tight balance between the utilization of fatty acids and other energy substrates is maintained (1). In metabolic heart disease myocardial ATP synthesis rate and free energy of ATP hydrolysis are decreased while ATP concentration is preserved (2). Metabolic remodeling and the decline of cardiac ATP production precede structural remodeling of the stressed heart and result from progressive maladaptation in substrate use and mitochondrial biogenesis and function (3, 4). Disrupted energy flux within the myocyte is recognized as a hallmark of cardiac failure (5). Metabolic remodeling not only disrupts cardiac energetics but also induces changes in cellular processes such as growth, redox homeostasis, and autophagy (6). Maladaptive changes in nutrient uptake, oxidation, and storage can lead to reduced energetic efficiency, ATP starvation, and ultimately cardiac dysfunction. This Research Topic is dedicated to articles (1) highlighting novel mechanisms that influence myocardial energy metabolism, (2) illustrating the role of cardiac metabolic pathways in health and disease, and (3) exploring translational avenues to target cardiac metabolism for the treatment of cardio-metabolic disorders.

In this Research Topic, Gopal et al. used a mouse model with cardiomyocyte-specific deficiency of pyruvate dehydrogenase to show that impaired myocardial glucose oxidation is sufficient to hinder diastolic, but not systolic heart function. Since obesity and diabetes-induced cardiomyopathy is often associated with reduced glucose oxidation, this study suggests that impairment of glucose oxidation *per se* can drive the development of cardiomyopathy and diastolic dysfunction and that this change in cardiac energy metabolism contributes to diabetic cardiomyopathy. Novel mechanisms underlying metabolic inflexibility and impaired glucose metabolism in diabetic cardiomyopathy are also highlighted in the mini-review article by Renguet et al. The authors summarized and interpreted our current understanding of the role of protein acetylation induced by the metabolism of non-glucosidic substrates in the impairment of insulin-stimulated glucose uptake in the heart during cardio-metabolic disease. While reduced myocardial glucose oxidation is implicated in the development of cardiomyopathy related to diabetes and obesity, excessive glucose utilization has also been associated with cardiac hypertrophy. In this Research Topic, Papur et al. discuss the role of protein kinase D in the regulation of cardiac glucose uptake and hypertrophy and delineate how protein kinase D isoforms influence cardiac energy metabolism, morphology, function, and hypertrophy. The relationship between loss of metabolic flexibility, mitochondrial ATP production, and heart failure is described in a review article by Karwi et al. examining preclinical and clinical studies. This review focuses on the alterations in fatty acid oxidation, carbohydrate metabolism, and ketone body metabolism in the heart in the setting of heart failure and illustrates the interplay among transcriptional regulation, post-translational modifications, and cytosolic/mitochondrial signaling kinases in regulating cardiac energy metabolism. The potential of modulating cardiac metabolism to enhance the efficiency of substrate utilization and mitigate cardiac dysfunction is discussed. While maladaptation of cardiac energy metabolism occurs in disease, including obesity, diabetes, and hypertrophy, physiological changes in cardiac substrate utilization are observed during exercise. Kolwicz provides a detailed overview of changes in glucose, fatty acid, ketone body, and amino acid metabolism in the heart during chronic exercise and discusses the exercise-induced adaptation of cardiac metabolism as a potential therapy for cardiac diseases such as hypertrophy.

Aberrant cardiac lipid metabolism is a hallmark of cardiometabolic diseases including diabetic cardiomyopathy and atherosclerosis. Yang et al. outline how myocardial autophagy of lipids, i.e., lipophagy, impacts lipid homeostasis in the heart muscle and atherosclerotic plaque and offer insight into the therapeutic potential of targeting autophagy for cardiometabolic diseases. A review article by Lal et al. shines a new light on vascular endothelial growth factor B and highlights its potential role in protecting against diabetic cardiomyopathy and heart failure by modulating cardiac metabolism and promoting cell survival. Saleme and Sutendra focus in their opinion article on heart failure triggered by chemotherapy-induced cardiotoxicity. They compare the metabolic signatures of the tumor and failing heart and offer directions to protect the heart during chemotherapy. The role of chronic low-grade inflammation in cardiovascular disease is examined by Ye and Ghosh. The authors provide their opinion on the usefulness of omega-3-polyunsaturated fatty acids compared to non-steroidal anti-inflammatory drugs in the prevention of myocardial inflammation. Because cardiac energy substrate metabolism is implicated in cardiac health and disease advancing our understanding of the complexities in the cardiac metabolic network will rationalize the utility of metabolic therapies targeting cardiovascular disease.

## Author's Note

PK is a Heart and Stroke Foundation of Canada New Investigator and TP is a Diabetes Canada Scholar.

## Author Contributions

All authors listed have made a substantial, direct and intellectual contribution to the work, and approved it for publication.

### Conflict of Interest Statement

The authors declare that the research was conducted in the absence of any commercial or financial relationships that could be construed as a potential conflict of interest.
